# To Those Who Have, More Will Be Given? Effects of an Instructional Time Reform on Gender Disparities in STEM Subjects, Stress, and Health

**DOI:** 10.3389/fpsyg.2022.816358

**Published:** 2022-02-21

**Authors:** Nicolas Hübner, Wolfgang Wagner, Jennifer Meyer, Helen M. G. Watt

**Affiliations:** ^1^Institute of Education, University of Tübingen, Tübingen, Germany; ^2^Hector Research Institute of Education Sciences and Psychology, University of Tübingen, Tübingen, Germany; ^3^Leibniz Institute for Science and Mathematics Education (IPN), Kiel, Germany; ^4^School of Education and Social Work, The University of Sydney, Sydney, NSW, Australia

**Keywords:** school reform, instructional time, gender disparities, STEM, achievement, motivation, stress, health

## Abstract

Educational reformers all around the globe are continuously searching for ways to make schools more effective and efficient. In Germany, this movement has led to reforms that reduced overall school time of high track secondary schools from 9 to 8 years, which was compensated for by increasing average instruction time per week in lower secondary school (Grades 5–10). Based on prior research, we assumed that this reform might increase gender disparities in STEM-related outcomes, stress, and health because it required students to learn similar content in less amount of time. Therefore, we investigated how the school time reform affected gender disparities at the end of upper secondary school between 2011 and 2013. Specifically, we considered representative data of the last two cohorts who completed lower secondary school before the reform (*N* = 2,405) and the first two cohorts after the reform (*N* = 2,413) from the National Educational Panel Study. Potential differences in gender disparities were investigated for upper secondary school outcomes of subject-specific standardized test performance, self-concept, and interest in mathematics, biology and physics, as well as outcomes of school-related stress and health. Overall, we found substantial disparities between girls and boys, which seemed to change little after the reform. Exceptions were the statistically significant gender × reform interactions for one stress dimension (Overload) and two health dimensions (Overburdening and Achievement-related fear) which increased for both boys and girls, but more strongly for girls.

## Introduction

The optimal amount of time needed to learn has a longstanding history of research and critical socio-political discussions (Pischke, 2007; Cuban, 2008). As summarized by Patall et al. (2010), whereas proponents suggest that more instructional time (e.g., in a given school year) improves student achievement, opponents have called this into question. In their systematic review, Patall et al. (2010) provided tentative evidence of the positive effects of increasing school time on student achievement, while reminding readers that much of what we currently know about this topic is based on weak designs. Considering further studies, findings on the effect of increasing instructional time on student achievement seem to be mixed, with some studies suggesting positive effects (e.g., Lavy, 2015; Andersen et al., 2016) and others finding zero or even negative effects (e.g., Allensworth et al., 2009; Nomi and Allensworth, 2009; Domina et al., 2015).

In contrast to these findings and intentions to increase instructional time, discussions regarding the optimal degree of time to learn went in a slightly different direction in Germany, where reforms from the past two decades were focused on making schools more efficient, for instance the “Gymnasium [high track secondary school] in 8 years”-reform (G8-reform). This reform aimed at reducing overall school time of high track secondary schools from 9 (G9) to 8 years (G8), which was compensated for by increasing average instruction time per week in lower secondary school (e.g., on average 3.69 additional hours per week each year; Homuth, 2017). Typically these reforms were implemented by either abolishing Grade 11 in upper secondary school or abolishing Grade 10 in lower secondary school (Kühn et al., 2013). Notably, in this study we focused on students from one German state (Baden-Württemberg). Here, overall instructional time per week was increased, while instruction time in STEM subjects remained largely comparable before and after the reform. Beyond this, further changes were implemented, which were required to increase instruction time per week, for instance new educational standards and school-specific curricula. Current research on the G8- reform is mixed in that some studies find student achievement to increase in lower secondary school (Huebener et al., 2017), whereas others find zero or negative effects on achievement, negative effects on stress levels and health, and delayed university enrollment of females (e.g., Büttner and Thomsen, 2015; Hübner et al., 2017a; Quis, 2018; Meyer et al., 2019; Marcus et al., 2020). Further studies are needed to investigate potential causes of these reported differences which may result from different samples (e.g., from different states) but also relate to the timeframe examined over which effects might accumulate or dissipate.

Although many school time studies focused STEM subjects, gender disparities, for instance on motivational outcomes or wellbeing, have been rarely investigated. This is surprising, because recent studies continue to find gender differences in STEM subjects (e.g., Watt, 2004; Else-Quest et al., 2010; Hübner et al., 2017b; Lazarides and Lauermann, 2019; Makarova et al., 2019; OECD, 2019) and on wellbeing (e.g., Hampel and Petermann, 2006; Moksnes et al., 2010; Salmela-Aro and Tynkkynen, 2012). Both motivation and wellbeing were found to be relevant for student achievement, aside from their importance in and of themselves (e.g., Widlund et al., 2018; Watt et al., 2019; Eccles and Wigfield, 2020; Wu et al., 2021). In addition, these constructs might also be affected by school reforms, as shown in prior studies (e.g., Hübner et al., 2017b; Marcus et al., 2020). It is consequently important to investigate whether girls may be disadvantaged relative to boys by the reform-induced changes, particularly regarding motivation and wellbeing. Therefore, in this study, we investigate gender disparities before and after the G8 school time reform in one German state (Baden-Württemberg) on an extended range of STEM-related outcomes beyond standardized test performance, such as subject-specific self-concept and interest in the subjects mathematics, biology and physics, and also include measures of school-related stress and health in the last year of secondary school.

## Gender and School Time

### Achievement, Gender, and School Time

Scarce evidence exists on gender disparities as a result of school time interventions or reforms. This is surprising for different reasons. First, gender equality is a central goal of all countries committed to human rights (United Nations General Assembly, 1948). Secondly, gender equality can contribute to economic growth (Altuzarra et al., 2021; Santos Silva and Klasen, 2021), particularly through increased participation in STEM jobs (Maceira, 2017; Hammond et al., 2020), which critically depend on achievement, self-concept, and course choices of STEM subjects in school (Updegraff et al., 1996; Parker et al., 2012; Watt et al., 2012, 2017; Schoon and Eccles, 2014. Referring to these arguments which underscore the relevance of monitoring effects of educational initiatives and reforms on gender disparities in general, it seems reasonable to believe that the G8-reform might specifically affect gender disparities in STEM. As girls and boys report different levels of self-concept and interest in math-intensive domains of STEM, which are central for subsequent achievement (e.g., Else-Quest et al., 2010; Hübner et al., 2017b,^2019^; Eccles and Wigfield, 2020; Wu et al., 2021), it is important to investigate if the reform-induced intensifications/compression in lower secondary school might affect gender disparities in STEM-related achievement and motivation.

Several studies found differential effects of instructional time reforms for high- and low-performing students. For instance, Nomi and Allensworth (2009) investigated the effect of the “Double-Dose” algebra reform in Chicago, which required Grade 9 students with test scores below the national median to participate in additional algebra courses. The authors found a stronger positive effect for students close to the median, compared with students who performed much lower. In the same vein, Huebener et al. (2017) found small and sometimes non-significant changes in mathematics and science achievement for lower deciles of the performance distribution in the course of the G8-reform in Germany, whereas effects were larger for higher deciles. To our knowledge, that study is the only one in which the potential effects of the G8-reform on gender disparities were examined in science, reading and mathematics achievement for Grade 9 students. Interestingly, the findings suggested no statistically significant differential effects on girls and boys in Grade 9. The timing of assessment is important to consider when interpreting results of different G8-studies, because G8 students in Grade 9 have had substantially more instructional time compared with G9 students in Grade 9. However, by the end of upper secondary school both cohorts have received a more comparable amount of instructional time.

In another study, Lavy (2015) reported that the treatment effect of increased school time was larger in higher performing countries, using PISA data. The author accounted for systematic differences between different countries by applying a country fixed-effects approach. These results provide tentative evidence of effect heterogeneity as a result of school time reforms, depending on students’ level of achievement.

Many school time studies and reforms focused on changes in STEM achievement of high and low performers, while gender disparities, for instance on motivational outcomes or wellbeing, have been rarely investigated. This constitutes an important limitation of many prior studies because gender disparities in STEM are well documented: The OECD (2019) reported a mathematics advantage for boys in 32 economies/countries (of 78; 14 economies/countries reported advantages for girls) and a science advantage for girls in 34 countries (of 78; 9 economies/countries reported advantages for boys). Notably, the differences were small on average (*d* = 0.05; ranging from *d* = 0.22 in Colombia to a non-significant difference of *d* = 0.01 in the Netherlands), and recent research suggests closings of these gaps, for instance in science achievement (e.g., Meinck and Brese, 2020). There are also meta-analyses that essentially found very small gender differences in math achievement but substantial variability across countries (e.g., Else-Quest et al., 2010). However, robust and systematic gender differences favor boys for math self-concept and interest in adolescence (e.g., Watt, 2004; Else-Quest et al., 2010; Frenzel et al., 2010; Nagy et al., 2010; Hübner et al., 2017b,^2019^; Widlund et al., 2018; Parker et al., 2020; Mejía-Rodríguez et al., 2021; Wu et al., 2021).

Probably most important in the context of this study, prior research using rich data from the end of German upper secondary school has provided evidence for substantial differences between boys and girls on a broad variety of mathematically intensive STEM outcomes, even after controlling for cognitive abilities. For instance, Hübner et al. (2019) found girls to have statistically significantly lower achievement in mathematics (*d* ≥ 0.45, *p* < 0.05) and physics (*d* ≥ 0.63, *p* < 0.05), compared to boys, whereas no such gender differences were found in biology. In addition, differences in mathematics in advantage of boys seem to be pronounced in Germany already by Grade 4 in elementary school (*d* = 0.18; Stanat et al., 2017).

### Self-Concept, Interest, Gender, and School Time

Women and men differ substantially in regard to their mathematical and mathematics-intensive STEM educational pathways and career aspirations (Watt et al., 2012, 2017; Schoon and Eccles, 2014; Lazarides and Lauermann, 2019; Makarova et al., 2019; Lazarides et al., 2020). This process has been referred to as the leaky STEM pipeline (Jacobs and Simpkins, 2005). Prior research has found that central to the choice of advanced course enrollments are students’ subject-specific achievement (Updegraff et al., 1996; Parker et al., 2012) and self-concept and values (Watt et al., 2012), even after controlling for prior achievement levels in the domain (Watt et al., 2017). These motivational variables have been linked not only to school enrollment but further to aspired educational and occupational pathways in mathematics and STEM subfields (Watt et al., 2012, 2017). Choosing advanced courses in high school constitutes a key factor for subsequent enrollment in STEM subjects at university (Ma and Johnson, 2008; Eccles and Wigfield, 2020; Lazarides et al., 2020). Thus, if a reform has differential effects on girls and boys (e.g., increases or decreases to their motivation), it is likely to affect subsequent decisions for or against related courses in high school or later on at university (e.g., Hübner et al., 2017b; Biewen and Schwerter, 2021).

This line of argumentation can be extended and implications can be derived more theoretically: Expectancy-value theory (Eccles, 1983; Eccles and Wigfield, 2002, 2020) outlines that key elements for choices are students’ expectations of success and task values and that both are influenced by prior achievement. Empirical evidence for this assumption can be found, for instance, in literature on the reciprocal effects model between self-concept and achievement (Marsh and Craven, 2006; Seaton et al., 2015). Self-concept is defined as students’ perceptions about their abilities, which develops via engagement with others (Shavelson et al., 1976; Marsh, 1990; Marsh et al., 2016). Task values, the other important set of variables to explain choices, consist of four components: intrinsic, attainment, utility, and cost values. Intrinsic value refers to students’ enjoyment when performing a specific task, attainment value refers to the personal importance a student attaches to a task, and utility value refers to its usefulness; researchers have combined attainment and utility values and referred to “importance value.” Costs, on the other hand, refer to the perceived negative consequences of task engagement, for example, effort or psychological and social costs (Watt et al., 2019).

Regarding subject-specific self-concept and interest, prior research suggests differences between girls and boys, which typically follow stereotypic patterns: Boys tend to report higher self-concept and interest in math-intensive STEM subjects compared to girls, whereas these effects are typically zero or in favor of girls in subjects such as biology (e.g., Denissen et al., 2007; Hübner et al., 2017b,^2019^; Watt et al., 2017, 2019; Parker et al., 2020; Mejía-Rodríguez et al., 2021). Therefore, if school time reforms force girls to learn similar content in less amount of overall time in subjects they are less interested in and in which they have lower perceptions of their own abilities (e.g., girls in math-intensive STEM subjects), this might even reinforce such less positive perceptions (e.g., Hübner et al., 2017b,^2019^). In addition, if the reforms differentially affect boys’ and girls’ STEM achievement this might also foster further disparities, for instance regarding students’ self-concept, as these variables are reciprocally related (e.g., Marsh and Craven, 2006; Arens et al., 2017; Wu et al., 2021).

### School-Related Stress, Health, Gender, and School Time

Other variables that are important to consider in the context of an intensified learning environment include students’ perceived stress and health. These variables might be particularly relevant in the context of increasing instructional time because it is intended that students spend more time with learning in school, which might lead to reduced or even too little leisure time to recover (Milde-Busch et al., 2010; Hübner et al., 2017a). As outlined in prior research, mental health is also associated with student achievement (e.g., Tuominen-Soini and Salmela-Aro, 2014; Fiorilli et al., 2017). For instance, Agnafors et al. (2021) found that students with mental health problems in very early years more often performed below grade level later on. Another study by Fiorilli et al. (2017) suggests that students’ burnout is highly relevant for student achievement, both directly and indirectly. The importance of considering wellbeing as a foundation for students’ aspirations was underscored in a study by Widlund et al. (2018) of Finnish students. Depending on the age group, the authors were able to identify either three (Grade 7) or four (Grade 9) latent profiles based on students’ attainment and self-concept in mathematics, their engagement, and three burnout subscales. They found that students with negative academic wellbeing had statistically significantly lower aspirations compared to thriving students. Interestingly, they found that girls were overrepresented in the negative academic wellbeing profile, which is in line with prior findings on gender disparities in school burnout (Salmela-Aro and Tynkkynen, 2012).

Beyond these studies, further research has produced evidence suggesting that girls generally do develop higher stress levels, compared with boys (e.g., Hampel and Petermann, 2006; Moksnes et al., 2010). Studies that have focused on investigating school stressors found schoolwork pressure to partly explain psychological complaints and psychosomatic pain (Hjern et al., 2008), and girls reported higher levels of performance-related stress at school (Moksnes et al., 2010). Finally, prior research provides evidence that increasing learning time might lead to more stress-related health problems (e.g., Marcus et al., 2020). Related to this, Quis (2018) investigated gender-specific differences between G8- and G9-students on school-related stress and health among students at the end of upper secondary school. She found considerable differences in school-related stress and mental health before and after the reform, mainly driven by girls (health) or boys and girls (stress). However, uncertainty exists whether such effects result from increases on a majority of stress facets (e.g., feelings of exhaustion, achievement-related overburdening, or not being able to recover in leisure time), or particularly on specific facets and not others.

## The Present Study

**FIGURE 1:**
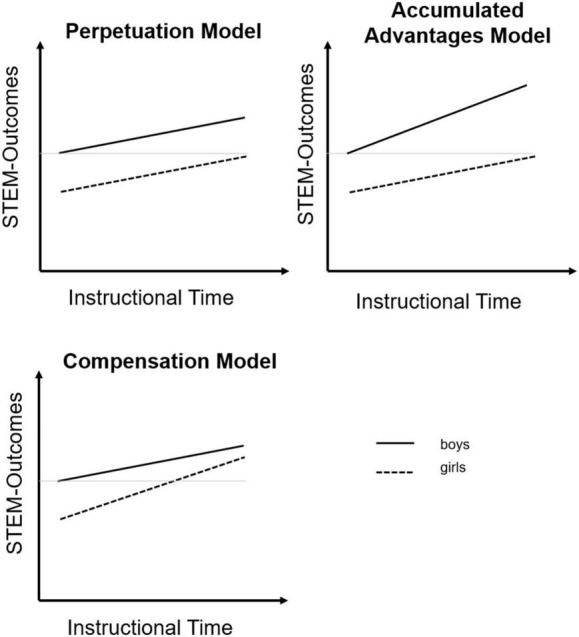
Hypothetical effects of the school time reform on gender disparities in math-intensive STEM subjects.

Based on our theoretical and empirical considerations above, three potential effects of the G8-reform on existing gender disparities in math-intensive STEM subjects can be derived, displayed in Figure 1. First, the *“perpetuation” model* would suggest no changes in disparities between boys and girls before and after the reform. This result pattern might be found, for instance, if the reform affected gender disparities in lower secondary school, where it was implemented, but these effects “washed out” by the end of upper secondary school, or if the reform-induced changes were too weak or equally affected boys and girls. Second, the “*accumulated advantages and disadvantages” model* would imply findings in the shape of the Matthew effect. This effect was first found by Merton (1968) and subsequently used by many researchers in educational, psychological and social scientific research to describe increasing disparities over time (e.g., for different ethnicities or students with different socio-economic backgrounds; e.g., Baumert et al., 2012). In the case of gender disparities, this effect would suggest that gender-specific advantages might increase (e.g., boys’ advantages over girls on achievement, self-concept, and task values in mathematics and physics), leading to overall widened disparities. Finally, the “*compensation” model* would imply that the disadvantaged group improves more over time, leading to smaller disparities after the reform. This effect would be found if girls benefit more from the reform, for instance because additional time is used to practice curricular content rather than to learn additional content (e.g., Hübner et al., 2017b).

Most of the cited literature above provides evidence for the accumulated advantages and disadvantages model, whereby school time reforms might particularly benefit higher performing students (Nomi and Allensworth, 2009; Lavy, 2015; Huebener et al., 2017), which would, in our case, imply widening gender-specific disparities on math-intensive STEM outcomes. Regarding STEM subjects, it is also important to consider hours per week in G8 vs. G9. Doing this, we found minor differences in officially reported hours in lower secondary school. Despite this, prior studies reported differences in student achievement between G8 and G9 students (Huebener et al., 2017; Hübner et al., 2017a). In our view, these findings underscore that it is important to not only consider subject-specific instructional time in school, but time spent on school-related purposes as a whole (e.g., Scheerens, 2014). For instance, even if instructional time were comparable in STEM subjects in G8 and G9, the overall instructional time per week in lower secondary school in G8 increased, which had an impact on the amount of time at home and students’ leisure time (Milde-Busch et al., 2010; Hübner et al., 2017a). Time at home constitutes a quite important predictor for school performance, for instance because students’ school-related engagements with parents can contribute to their learning (Berkowitz et al., 2015), investing time in homework might improve student achievement (Rawson et al., 2017), and leisure time can be used for addressing specific learning gaps, preparing for exams outside from school, or to recover from school-related stress (Milde-Busch et al., 2010). Further, girls were found to invest more time at home for school-related purposes (Wagner et al., 2008), which might also explain potential differential effects of the G8-reform. From this perspective, even if instructional time in STEM subjects remains comparable, if students have to invest more time, on average, in formal schooling and have less time for self-paced learning, learning activities at home, or relaxation, this might have detrimental effects on their achievement and wellbeing.

As outlined above, disparities between boys and girls were inconsistent and small at most in mathematics achievement, substantially larger in math self-concept and non-existent in science (Watt, 2004; Else-Quest et al., 2010; Watt et al., 2012;OECD, 2019). Based on this, we expect zero or very small effects on math or math-intensive STEM achievement, larger effects on math-intensive STEM self-concept and interest, but null effects for biology. Regarding stress and health, it seems reasonable to believe that the reform might be perceived as more demanding by girls compared to boys, which might produce larger differences between boys and girls after the reform. As girls report higher levels of burnout and stress than boys (e.g., Salmela-Aro and Tynkkynen, 2012; Widlund et al., 2018), increasing demands of the learning environment might particularly be harmful for them. Prior research has (on average) found larger disparities between boys’ and girls’ school-related stress levels after the reform (Quis, 2018), but has not yet explored whether average differences might mask differences on specific stress facets but not others. We will extend findings based on unidimensional models to obtain a nuanced understanding of gender-specific reform effects on different dimensions of stress and health.

## Materials and Methods

### Description of the Study and Sample

We used data from the Additional Study Baden-Württemberg (Blossfeld et al., 2011) from the National Educational Panel Study (NEPS; Scientific Use File 3.2.0). The dataset contains representative data for Baden-Württemberg, assessed from four different cohorts in the final semester of upper secondary school. Two cohorts completed German lower secondary school before the reform and two completed it after the reform. We compared outcomes of these cohorts assessed at the end of upper secondary school (G9: Grade 13 or G8: Grade 12). This design is typically referred to as a cohort control design (Shadish et al., 2002). Overall, students from 44 high track upper secondary schools participated in the study: Cohort 1 (before the reform): *n* = 1,226 (55% girls); Cohort 2 (before the reform): *n* = 1,179 (55% girls); Cohort 3 (after the reform): *n* = 1,205 (56% girls); Cohort 4 (after the reform): *n* = 1,208 (55% girls). Before the reform, students graduated after 9 years of high track upper secondary school, whereas after the reform students graduated after 8 years. The first cohort of students graduated in 2011, the second (Grade 13) and third (Grade 12) in 2012, and the fourth in 2013. Notably, in Baden-Württemberg, Grade 11 was abolished to implement the G8-reform (Kühn et al., 2013). Data were collected in the final semester of the last year of upper secondary school. Students in Germany are required to spend at least 265 h per week each year in school. This means that G9 students are required to spend on average 265/9 = 29.44 h per week each year in school, whereas G8 students are required to spend 265/8 = 33.13 years per week each year in school, reflecting a difference of 3.69 additional hours that students in G8 are required to spend per week in school. Overall, cumulated mandatory hours were 11 h higher for G8 students from grade 5 to grade 6, and 16 h higher for G8 students from grade 7 to grade 10 in Baden-Württemberg (Homuth, 2017).

### Instruments

In all cohorts, identical instruments were administered to assess subject-specific standardized achievement, self-concept, and interest in the subjects mathematics, biology and physics, as well as to assess school-related stress and health. The questionnaire is available in the NEPS data center^1^.

#### Standardized Test Performance

Comprehensive information on these tests and different quality indicators can be found in the scaling reports of the National Educational Panel Study (Duchhardt, 2015; Hübner et al., 2016a,b). The mathematics test was based on 20 items from the four areas of quantity, space/shape, change/relationships, and data/chance (Duchhardt, 2015). The biology test consisted of 60 items from the areas of cytology/anatomy/metabolism, information processing/characteristics/immunology, genetics/development biology, ecology, and systematics/evolution (Hübner et al., 2016a). Finally, physics achievement was assessed using 41 items from nine different areas, for instance electrical fields and interdependency, waves, and optics (Hübner et al., 2016b). In our sample, the reliability of the weighted likelihood estimator (WLE; Adams, 2005) was *Rel.* = 0.70 for the math test, *Rel.* = 0.61 for the physics test, and *Rel.* = 0.73 for the biology test. As outlined below, latent variable models were specified to adequately address their measurement error.

#### Subject-Specific Self-Concept

Subject-specific self-concept was assessed using four items from the translated Self-Description Questionnaire III (Marsh and O’Neill, 1984) for each of the subjects mathematics, biology and physics. For example, students were asked to rate their agreement to: “I have never done well in mathematics” or “I am good at mathematics” on a 4-point rating scale from 1 (*does not apply at all*) to 4 (*completely applies*). Negatively formulated items were reverse coded. Cronbach’s α for students’ self-concept was α = 0.94 for mathematics, α = 0.91 for biology, and α = 0.94 for physics.

#### Subject-Specific Interest

Subject-specific interest was assessed using four items based on the expectancy-value framework (Eccles, 1983; Eccles and Wigfield, 2002) for each of the subjects mathematics, biology, and physics. Items were comparable to those from prior German large-scale studies (Trautwein et al., 2006, 2010). For instance, students were asked to rate their agreement to: “It is important for me personally to be good at mathematics” or “Math is just exciting for me” on a 4-point rating scale from 1 (*does not apply at all*) to 4 (*completely applies*). Negatively formulated items were reverse coded. Cronbach’s α for students’ interest was α = 0.82 for mathematics, α = 0.87 for biology, and α = 0.90 for physics.

#### School-Related Stress

School-related stress was assessed using 15 items (Hübner et al., 2017a). Example items are: “Sometimes I have trouble falling asleep because problems from school are on my mind,” “Even during my free time I think about troubles at school,” or “Pressure at school is too high” (see Supplementary Table 1 for a full list of items). Students were asked to answer these items on a 4-point rating scale ranging from 1 (*completely disagree*) to 4 (*completely agree*). The stress scale constitutes an instrument which was developed by the NEPS (including internal review cycles), which has a specific focus on school-related stress. Both instruments were also administered in the NEPS Thuringia study (Blossfeld et al., 2011). Negatively formulated items were reverse coded. Reliability of the scale was high (Cronbach’s α = 0.91).

#### Health

Students’ health was measured by asking them to rate how often they experienced 26 different health problems on a rating scale from 1 (*never*) to 4 (*more than 6 times during the last 6 weeks*), respectively (Bergmüller, 2007). Among others, health problems such as “headaches,” “sleep disturbances,” “vomiting,” or “feelings of inner emptiness” were assessed (see Supplementary Table 2 for a full list of items). There are further studies, which administered comparable health items, particularly in the field of medical science (e.g., Milde-Busch et al., 2010), but also beyond (Bergmüller, 2007). The health scale was administered in prior cycles of the PISA study (Bergmüller, 2003). Reliability of the scale was high (Cronbach’s α = 0.92).

In examining these outcomes, we controlled for a variety of covariates in the adjusted models. These were immigration background (i.e., students with at least one parent born abroad), number of available books at home, highest international socioeconomic index in the family (HISEI), non-verbal cognitive skills (i.e., perceptual speed and reasoning; Haberkorn and Pohl, 2013), and whether students had repeated a class. In addition, we controlled for the course level (advanced, basic, or de-selection) when investigating differential effects on standardized test performance. An overview on course enrollment by gender and subject is given in Supplementary Table 5. Notably, there were no gender differences in math enrollment, as all students are mandated by law to enroll in advanced mathematics courses (4 h per week), whereas differences were most visible in physics, where only 8.7% of girls were enrolled in advanced courses, compared to 29.9% of boys.

### Statistical Analysis

The main analysis proceeded in two steps. First, we estimated multiple-group models for the eight different groups (4 cohorts × gender) in M*plus* 8.6 (Muthén and Muthén, 1998–2017). We did this separately for standardized test achievement, self-concept, interest, school-related stress, and health. For achievement, we used multidimensional (multiple-group) item response theory (IRT) models (see Jöreskog and Goldberger, 1975; Hübner et al., 2020). For the remaining constructs traditional structural equation models (SEMs) were applied. Prior work offered clear guidance on how to define measurement models for the achievement measures, self-concept, and interest (e.g., Marsh, 1992; Eccles and Wigfield, 2002; Duchhardt, 2015; Hübner et al., 2016a); this was not the case for the instruments used to assess students’ stress and health which were typically analyzed as a single aggregate score and not with multidimensional models (e.g., Bergmüller, 2003; Hübner et al., 2017a; Quis, 2018). In this study, we utilized a data-driven procedure to explore the underlying factor structure of stress and health items using exploratory structural equation models (ESEMs). As outlined by Marsh et al. (2014), ESEMs combine useful features of exploratory and confirmatory factor analysis (EFA/CFA) such as confirmatory tests of factor structures and associations between different latent factors, and they allow small cross-loadings. For school-related stress and health we performed ESEMs with geomin rotated factor loadings in a multiple group framework. To identify the most adequate solution, we first specified different (single-group) ESEM models with an increasing number of latent factors, before running ESEMs in a multiple group framework with the eight groups (gender × cohort). Models were constrained to test strong factorial/scalar measurement invariance, which is required to meaningfully compare latent means across groups. To judge model fit, we considered the Comparative Fit index (CFI), the Tucker-Lewis index (TLI), the Root Mean Square Error of Approximation (RMSEA), and the Standardized Root Mean Square Residual (SRMR). Based on prior research (MacCallum et al., 1996; Hu and Bentler, 1999; Yu, 2002), we considered the following cutoffs to indicate good model fit: CFI and TLI ≥ 0.95, SRMR and RMSEA ≤ 0.05.

Using these models, we compared the means or—for models with covariates—intercepts of the latent outcomes between the resulting groups using the delta method (Oehlert, 1992) by applying the MODEL CONSTRAINT option in M*plus*. Statistically significant differences between the specific group differences constitute interaction effects. We estimated (a) gender differences in G9 cohorts and (b) gender differences in G8 cohorts, and one interaction effect: (c) the difference between a and b (reform × gender). We specified unadjusted models without covariates and adjusted models including covariates (e.g., cognitive abilities, socioeconomic background; see “Instrument” section) to check the robustness of our results. To better interpret our findings, results were transformed into a metric with an overall *M* = 500 and *SD* = 100 for achievement and to a metric with an overall *M* = 50 and *SD* = 10 for the remaining constructs, using the pooled variance of the latent variables from the unadjusted models. For consistency, we report two-sided *p*-values throughout, although prior studies suggest a directional hypothesis for stress, health, and math-intensive STEM self-concept in disadvantage of girls. We therefore interpret one-sided *p*-values to judge statistical significance for those constructs (one-sided *p*-value = two-sided *p*-value/2). For all other outcomes, no consistent directional hypothesis could be derived from the literature. All models were specified using full information maximum likelihood estimation (FIML; Enders, 2010), robust standard errors (McNeish et al., 2017), and survey weights.

## Results

### Preliminary Analysis

First, we inspected descriptive statistics. As shown in Table 1, overall, differences between the two cohorts were small. Only with regard to perceptual speed, students in G9 scored slightly higher. Further, students in G8 repeated classes slightly less often than students in G9. This resulted from a generally low repetition rate due to a specific feature of the reform implementation: If students from the last G9 cohort were required to repeat a grade, they had to move from, for instance, the end of grade 10 to the beginning of grade 9, because the respective grade 10 cohort in G8 would have already been ahead of the grade 10 in G9, which the student should repeat (due to the additional hours per week in lower secondary school). These differences were controlled for in the adjusted models as outlined below.

**TABLE 1:** Descriptive statistics on central covariates before and after the reform.

	G9		G8			
	*n* = 2.405		*n* = 2.413			
Variable	*M*	*SD*	*M*	*SD*	*ES*	*p*
Immigration background (1 = yes)	0.23	0.42	0.22	0.41	1%	0.349
Books at home	4.72	1.24	4.73	1.25	–0.01	0.858
HISEI	58.16	15.42	58.41	15.50	–0.02	0.653
Perceptual speed	65.32	11.41	64.98	11.96	0.03	0.660
Reasoning	10.80	1.26	10.71	1.27	0.07	0.023
Class repeater (1 = yes)	0.10	0.30	0.06	0.24	4%	<0.001

*Descriptive statistics were estimated using full information maximum likelihood estimation, cluster-robust standard errors, and survey weights. HISEI = highest international socioeconomic index in the family. ES = Effect size. We used Cohen’s d (Cohen, 1988) for continuous variables, which was estimated as M_G9_-M_G8_ divided by the pooled SD, and differences in percentage points for dichotomous variables. Please also see Hübner et al. (2017a) and Quis (2018) for additional tests of potential selectivity and representativeness and comparisons of differences on covariates across different cohorts. Additional information on the estimation of the survey weights can be found in Schönberger and Aßmann (2014).*

### Gender-Specific Differences Before and After the Reform

Next, we inspected gender-specific differences. As visible from Table 2, we found substantial differences between girls and boys, both before and after the reform.

**TABLE 2:** Unadjusted gender-disparities before and after the reform on standardized test performance, subject-specific self-concept and interest, and school-related stress and health.

	*b*_*G*9_	SE	*p*	*b*_*G*8_	SE	*p*	Δ *b*	SE	*p*
**Standardized test performance**									
Biology	**18.84**	5.80	0.001	**18.61**	6.10	0.003	0.23	8.85	0.975
Mathematics	**79.23**	5.75	<0.001	**70.60**	5.31	<0.001	8.63	7.64	0.254
Physics	**91.85**	6.05	<0.001	**92.64**	5.19	<0.001	−0.79	6.92	0.915
**Subject-specific self-concept**									
Biology	0.00	0.54	0.999	−0.07	0.42	0.870	0.07	0.67	0.917
Mathematics	**4.06**	0.48	<0.001	**4.82**	0.46	<0.001	−0.76	0.64	0.231
Physics	**6.55**	0.48	<0.001	**7.02**	0.48	<0.001	−0.47	0.68	0.488
**Subject-specific interest**									
Biology	−0.59	0.59	0.320	−1.14	0.53	0.030	0.55	0.72	0.446
Mathematics	**1.89**	0.56	0.001	**2.76**	0.50	<0.001	−0.87	0.80	0.268
Physics	**6.48**	0.54	<0.001	**5.53**	0.64	<0.001	0.95	0.82	0.238
**School-related stress**									
Difficulties to relax	**−7.34**	0.69	<0.001	**−8.58**	0.71	<0.001	1.24	0.86	0.149
Exhaustion	**−5.03**	0.63	<0.001	**−5.60**	0.53	<0.001	0.57	0.72	0.422
Overload	**−2.00**	0.51	<0.001	**−3.61**	0.75	<0.001	**1.61**	0.73	0.027
Malaise	**2.60**	0.93	0.005	1.49	1.09	0.171	1.11	0.75	0.134
Alignment issues	**−3.15**	0.67	<0.001	**−4.00**	0.77	<0.001	0.85	0.75	0.256
**Health**									
Overburdening	**−3.69**	0.74	<0.001	**−5.01**	0.70	<0.001	**1.32**	0.74	0.076
Achievement-related fear	**−6.12**	0.72	<0.001	**−7.81**	0.70	<0.001	**1.69**	0.64	0.008
Diverse symptoms	**−13.05**	2.56	<0.001	**−14.08**	2.52	<0.001	1.03	1.00	0.301
Uneasiness	−1.77	2.11	0.352	−2.25	1.90	0.285	0.49	0.69	0.476
Depressive symptoms	**−3.60**	0.66	<0.001	**−4.63**	0.73	<0.001	1.02	0.65	0.119
Gastrointestinal issues	**−3.62**	1.48	0.004	**−4.01**	1.11	0.012	0.39	0.72	0.492

*b _G9_ = Gender differences before the reform; b _G8_ = Gender differences after the reform. Positive values indicate higher values for boys. Δb = Difference of gender differences before (G9) minus after (G8) the reform. The metric of the latent variable was transformed to M = 500 and SD = 100 for standardized test performance and to M = 50 and SD = 10 for all other outcomes using pooled means and standard deviations. Statistically significant differences (p < 0.05) are printed in bold. Regresson coefficients (b’s) are based on group mean differences in a multiple group model. Two-sided p-values are reported. In cases where we had a directional hypothesis based on prior literature (e.g., higher stress scores of girls), one-sided p-values should be calculated/interpreted, which can be calculated by dividing the reported two-sided p-value by 2.*

#### Standardized Test Performance

Regarding standardized test performance, boys were found to score statistically significantly higher than girls before the reform in biology (*b* = 18.84, *p* = 0.001), in mathematics (*b* = 79.23, *p* < 0.001), and in physics (*b* = 91.85, *p* < 0.001). Differences were smaller in biology and substantially larger in mathematics and physics, and these differences remained equally pronounced after the reform. After the reform, the respective differences amounted to *b* = 18.61 (*p* = 0.003) in biology, *b* = 70.60 in mathematics (*p* < 0.001), and *b* = 92.64 points (*p* < 0.001) in physics. Notably, differences between gender disparities from before and after the reform (i.e., the gender × reform interaction effect) were not statistically significant for any standardized test performance. This coefficient amounted to Δ*b* = 0.23 points (*p* = 0.975) in biology, Δ*b* = 8.63 (*p* = 0.254) in mathematics, and Δ*b* = −0.79 (*p* = 0.915) in physics. These results suggest that differences between girls and boys were generally large on these standardized test outcomes before the reform and remained comparably large after the reform, consistent with the perpetuation model (see Figure 1).

#### Subject-Specific Self-Concept

With regard to subject-specific self-concept, we found a slightly different picture. Here, no statistically significant differences between boys and girls were found for biology, before (*b* = 0.00, *p* = 0.999) or after the reform (*b* = −0.07, *p* = 0.870). Regarding mathematics, girls and boys differed statistically significantly before the reform (*b* = 4.06, *p* < 0.001) and after the reform (*b* = 4.82, *p* < 0.001), with boys having higher self-concept scores. The differences in gender disparities before vs. after the reform did not reach statistical significance (Δ*b* = −0.76, *p* = 0.231). Finally, regarding physics, a similar picture as in mathematics emerged. Boys had higher scores before (*b* = 6.55, *p* < 0.001) and after (*b* = 7.02, *p* < 0.001) the reform, and these differences did not change (Δ*b* = −0.47, *p* = 0.488).

#### Subject-Specific Interest

Next, we had a closer look at the results for subject-specific interest. The results were fairly similar to those for subject-specific self-concept, however, gender differences were less pronounced in mathematics. Here differences amounted to 1.89 points (*p* = 0.001) before the reform and 2.76 points after the reform (*p* < 0.001). The reform × gender interaction effect did not reach statistical significance (Δ*b* = −0.87, *p* = 0.268). In summary, the results for achievement test performance, subject-specific self-concept, and subject-specific interest provided evidence in support of the perpetuation model.

#### School-Related Stress

Subsequently, we investigated potential differences for school-related stress. To do this, we first fitted a series of ESEM models with an increasing number of latent factors. The solution to first reach adequate model fit (CFI and TLI ≥ 0.95 and RMSEA and SRMR ≤ 0.05) was a model with six factors; however, one factor had substantial loadings only on the (reverse-coded) negatively worded items, while the loadings of these items on all other factors were small (all ≤ 0.06 for t5m and ≤ 0.02 for t5n). Also considering findings from prior studies on challenges of considering negatively worded items of instruments (e.g., DiStefano and Motl, 2006; van Sonderen et al., 2013; Zhang et al., 2016), we decided to drop the two reverse-scored items, which resulted in a more parsimonious five-factor multiple group model [unadjusted model: χ^2^(520) = 833.378, *p* < 0.001, CFI = 0.99, TLI = 0.98, RMSEA = 0.03, SRMR = 0.03]. From a substantive perspective, this model was comparable to the model with six factors but did not include the factor for the negatively worded items. As a robustness check, we also specified a model in which we predicted the previously dropped (reverse coded) items t5n and t5m by the five factors, a reform dummy variable, gender, and the interaction term reform × gender. Our findings showed that, after conditioning on the five factors, none of the remaining variables was statistically significantly associated with the t5n or t5m variable. Therefore, it seems unlikely that dropping the two negatively worded items had a substantial impact on our main research question. The five factors were given names based on their loading patterns (see Supplementary Table 3): (1) Difficulties to relax, (2) Exhaustion, (3) Overload, (4) Malaise, and (5) Alignment issues. As is visible in Table 2, we found statistically significant differences between boys and girls on all factors in G9 (all *p*s ≤ 0.005) and on all factors besides Malaise (*p* = 0.171) in G8 (all *p*s < 0.001). Whereas these differences generally suggested higher stress levels for girls on four of five factors (Difficulties to relax, Exhaustion, Overload, and Alignment issues), boys in G9 reported having more issues on the Malaise factor. Finally, we found a statistically significant gender × reform interaction effect on the Overload factor (Δ*b* = 1.61, *p* = 0.027). This factor had its highest loadings on items such as “Pressure at school is too high” or “I consider the requirements at school in general as stressful.” The interaction effect indicated that the difference between boys and girls on this factor was larger in G8 than G9. Further explorations revealed that it was strongly driven by larger overload stress levels for girls in G8 vs. G9 (Δ*b* = 5.21, *p* < 0.001), compared to boys (Δ*b* = 3.60, *p* < 0.001).

#### Health

For health, we found an ESEM model with six factors to reach the cutoff values for model fit as outlined above [unadjusted model: χ^2^(2,452) = 4,041.463, *p* < 0.001, CFI = 0.95, TLI = 0.95, RMSEA = 0.03, SRMR = 0.04]. The six factors were given names based on their loading patterns (see Supplementary Table 4): (1) Overburdening, (2) Achievement-related fear, (3) Diverse symptoms, (4) Uneasiness, (5) Depressive symptoms, and (6) Gastrointestinal issues. The results pointed in the same direction as for stress: Girls tended to have statistically significantly more health issues on all six health factors, although the difference on the Uneasiness factor between boys and girls in G9 and G8 cohorts was not statistically significant (see Table 2). The largest difference was found on the Diverse symptoms factor, which had as its three highest loadings the indicators “Headaches,” “Bad dreams,” and “Stomach ache” (G9: *b* = −13.05, *p* < 0.001; G8: *b* = −14.08, *p* < 0.001).

**FIGURE 2:**
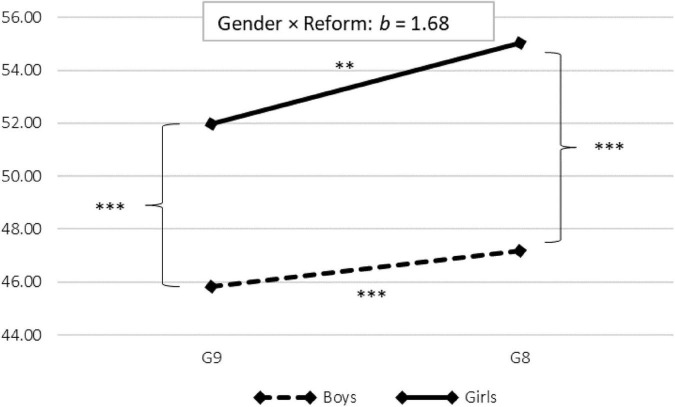
Gender-specific interaction effect for achievement-related fear. Based on findings reported in Table 3. ^***^p < 0.001. ^**^p < 0.01.

For health, we found two statistically significant gender × reform interaction effects on the factors Overburdening (highest loadings for “Difficulty concentrating,” “Tiredness, fatigue,” and “Easily irritable”) and Achievement-related fear (“Feeling that excessive demands are being made of me,” “Fear of going to school,” “Fear that it’s all getting too much”). For Overburdening, this interaction effect amounted to Δ*b* = 1.32 (*p* = 0.076 [*p*_*one*–*sided*_ = 0.038]), whereas for Achievement-related fear, it amounted to Δ*b* = 1.69 (*p* = 0.008). The interaction effect for Achievement-related fear is displayed in Figure 2, which increased more for girls than boys following the reform.

**TABLE 3:** Adjusted gender-disparities before and after the reform on standardized test performance, subject-specific self-concept and interest, and school-related stress and health.

	*b*_*G*9_	SE	*p*	*b*_*G*8_	SE	*p*	Δ *b*	SE	*p*
**Standardized test performance**									
Biology	**19.29**	6.41	0.002	**19.98**	5.80	0.001	–0.69	8.85	0.943
Mathematics	**78.57**	5.53	<0.001	**68.44**	4.76	<0.001	10.13	6.86	0.142
Physics	**89.90**	4.61	<0.001	**90.77**	5.19	<0.001	–0.86	5.62	0.872
**Subject-specific self-concept**									
Biology	−−0.01	0.54	0.992	−−0.19	0.42	0.650	0.18	0.68	0.783
Mathematics	**4.02**	0.44	<0.001	**4.45**	0.42	<0.001	–0.42	0.59	0.468
Physics	**6.55**	0.43	<0.001	**7.01**	0.44	<0.001	–0.46	0.62	0.467
**Subject-specific interest**									
Biology	−−0.51	0.57	0.369	−−0.96	0.56	0.088	0.45	0.74	0.550
Mathematics	**1.94**	0.53	<0.001	**2.37**	0.50	<0.001	–0.43	0.75	0.564
Physics	**6.55**	0.51	<0.001	**5.43**	0.59	<0.001	1.12	0.78	0.147
**School-related stress**									
Difficulties to relax	−**7.42**	0.71	<0.001	**−8.40**	0.81	<0.001	0.98	0.90	0.271
Exhaustion	−**5.08**	0.66	<0.001	−−**6.09**	0.49	<0.001	1.01	0.72	0.161
Overload	−**2.05**	0.51	<0.001	−−**3.68**	0.70	<0.001	**1.63**	0.69	0.017
Malaise	**2.82**	0.97	0.004	1.48	1.10	0.176	1.34	0.87	0.123
Alignment issues	−**3.29**	0.67	<0.001	−−**4.14**	0.82	<0.001	0.86	0.77	0.265
**Health**									
Overburdening	−**3.63**	0.70	<0.001	−−**4.90**	0.74	<0.001	**1.28**	0.75	0.089
Achievement-related fear	−**6.17**	0.69	<0.001	−−**7.85**	0.72	<0.001	**1.68**	0.65	0.010
Diverse symptoms	−**12.36**	2.66	<0.001	−−**13.31**	2.84	<0.001	0.95	0.95	0.586
Uneasiness	−−2.19	2.39	0.361	−−2.71	2.56	0.288	0.53	0.78	0.498
Depressive symptoms	−**3.39**	0.66	<0.001	−−**4.31**	0.62	<0.001	0.91	0.59	0.125
Gastrointestinal issues	−**3.46**	0.92	<0.001	−−**3.86**	0.93	<0.001	0.39	0.64	0.541

*b _G9_ = Gender differences before the reform; b _G8_ = Gender differences after the reform. Positive values indicate higher values for boys. Δb = Difference of gender differences before (G9) minus after (G8) the reform. The metric of the latent variable was transformed to M = 500 and SD = 100 for standardized test performance and to M = 50 and SD = 10 for all other outcomes using pooled means and standard deviations. Statistically significant differences (p < 0.05) are printed in bold. Regression coefficients (b’s) are based on group mean differences in a multiple group model. Two-sided p-values are reported. In cases where we had a directional hypothesis based on prior literature (e.g., higher stress scores of girls), one-sided p-values should be calculated/interpreted, which can be calculated by dividing the reported two-sided p-value by 2. Covariates that were considered for adjustment can be found in the Instrument section. For achievement, explained variance of the latent variables ranged between 12 and 44% (M = 26%), for self-concept between 1 and 15% (M = 6%), for interest between 1 and 11% (M = 4%), for stress between 0 and 6% (M = 2%), and for health between 0 and 13% (M = 5%). Note that when excluding course level as a covariate, results remained comparable regarding the size of estimates. In addition, in these models all SEs in the adjusted models were smaller compared to the unadjusted models. Please be aware that when estimating results for stress and health, we applied exploratory SEMs, which led to slightly different measurement models (i.e., differences in factor loadings), when additional variables (e.g., covariates) were considered and this explains differences in SEs between the adjusted and unadjusted solution. Although the general loading pattern (see Supplementary Tables 3, 4) remained similar in adjusted and unadjusted models, SEs should not be directly compared across these two solutions, because they refer to slightly different latent variables.*

Finally, we compared results from the unadjusted and adjusted models (see Table 3), in which we controlled for further covariates such as cognitive abilities and socioeconomic background. Overall, we did not find substantial differences between the two solutions, in terms of statistical significance or the direction or size of coefficients (see Tables 2, 3). Our results for achievement, self-concept, and interest provide tentative evidence in line with the perpetuation model, whereas our findings for stress and health are more in line with the accumulated advantages/disadvantages model (see Figure 1).

## Discussion

In this study, we investigated the effects of the G8-reform on gender disparities in STEM achievement, self-concept, and interest, as well as school-related stress and health. To do this, we compared data of four successive student cohorts, two from before the reform and two from afterward. Specifically, the reform changed the overall school time of high track secondary schools from 9 to 8 years, which was compensated for by increasing average instruction time per week in lower secondary school (Grades 5–10 in Germany).

Taken as a whole, this study has brought to light several important findings. First of all, we found substantial gender disparities in favor of boys at the end of upper secondary school on the respective STEM outcomes. Disparities were pronounced regarding the achievement in mathematics and physics and substantially smaller in biology (e.g., only 1/4 of the size of mathematics achievement). This is an important finding and underscores that gender-related disparities reported in prominent large-scale studies of students in Grade 9 might not reflect actual disparities at the end of upper secondary school in Germany, a key stage in the education system, right before students enroll in university. It also reflects previously articulated heterogeneity in disparities across countries (OECD, 2019; Parker et al., 2020) and underlines the importance of more closely considering disparities at different time points in the education system in future studies.

Second, our findings show that a unidimensional perspective on school-related stress and health masks result patterns that appeared when investigating the constructs at a more fine-grained level of underlying dimensions. A five-factor multiple group ESEM model constituted multidimensional school-related stress, and a six-factor model constituted health. Although the patterns were more or less consistent and in disadvantage of girls, there were exceptions, for instance regarding the Malaise aspect of school-related stress where we found disadvantages for boys, and on the Uneasiness aspect of health where we found no statistically significant differences.

Finally, and most important in the context of this study, the gender disparities evident before the reform seemed to perpetuate after the reform for STEM-related standardized test performance, self-concept, and interest. For school-related stress and health we found some statistically significant gender × reform interaction effects more in line with an accumulated advantages/disadvantages model (see Figure 1; i.e., on the Overload dimension of stress, and the Overburdening and Achievement-related fear dimensions of health). This suggests that although both girls and boys reported substantially higher stress levels and lower health after the reform, the increase or decrease, respectively, was somewhat larger for girls than boys, at least on some stress and health facets.

### Gender Disparities and the School Time Reform

As outlined above, we found large disparities between girls and boys at the end of upper secondary school on STEM-related outcomes. In most cases, these disparities followed stereotypical patterns: Overall, girls performed less well on standardized tests in math-intensive STEM subjects. In addition, girls reported lower self-concept and interest than boys in mathematics and physics, whereas there were no significant gender-related disparities in biology. When integrating our findings into the theoretical model (see Figure 1), we can summarize that in most cases we found evidence for the perpetuation model. Disparities before the reform on the respective outcomes were pronounced, and these differences did not change much after the reform. Our findings extend prior findings in three regards: They are based on a later period in the education system (end of upper secondary school, right before the transition to university), a broadened set of outcomes, and a more fine-grained investigation of school-related stress and health.

As we outlined in the theoretical background, several prior studies had suggested treatment effect heterogeneity for high and low achievers (e.g., Nomi and Allensworth, 2009; Lavy, 2015; Huebener et al., 2017), which is why we expected we would find a pattern of results in line with the accumulated advantages model (Figure 1) for STEM outcomes. However, aside from few stress and health facets, we did not find any changes when comparing gender disparities before and after the reform. This might have had different causes—for instance, students in our sample were older at the end of secondary school, compared with students in the reviewed studies. Therefore, our sample might constitute a positive selection of higher performing students as some lower performing students might have dropped out before or in early upper secondary school or might have switched to vocational upper secondary schools, where this reform was not implemented. This might have led to smaller gender differences in upper secondary school than before, in lower secondary school. Further, the major changes of the G8-reform happened in lower secondary school, whereas upper secondary school remained largely unaffected. Therefore, potential interaction effects on STEM outcomes might already have “washed out” by the end of upper secondary school. Most importantly, when comparing differences between G8 and G9 students’ average weekly hours spent in STEM courses, we found negligible differences. This means, that changes in subject-specific instructional time might have been a too small and a central factor for why we did not find any differences on STEM related outcomes. However, this would not explain previously found reform-specific differences between G8 and G9 students for instance in Biology (Hübner et al., 2017a).

In contrast to perpetuating disparities on STEM outcomes after the reform, our study revealed some statistically significant interaction effects on school-related stress and health. Importantly, both girls and boys tended to report more school-related stress and health issues after the reform. However, we did not find interaction effects on all stress and health dimensions, but only on those more related to school, namely the Overload dimension of school-related stress, and the Overburdening and Achievement-related fear dimensions of health. Compared to the perpetuating subject-specific results outlined above, these findings are slightly more in line with the proposed accumulated (dis)advantages model: On average, all students (girls and boys) tended to report higher stress/poorer health after the reform, but particularly those students who were more stressed/had lower health scores before the reform seemed to experience higher school-related stress and poorer health afterward, at least on stress and health facets more closely related to school. These results are in line with prior findings that girls report lower wellbeing scores than boys (e.g., Moksnes et al., 2010; Salmela-Aro and Tynkkynen, 2012; Tuominen-Soini and Salmela-Aro, 2014) and reflect findings from prior studies that students might perceive the remaining leisure time to be too limited to recover from school-related stress (Milde-Busch et al., 2010). The higher average workload per week in lower secondary school as a result of the G8-reform might have been one driver of the unevenly higher stress for girls after the reform. Other potentially relevant stressors than the higher workload could have included longer school days, the abolishment of Grade 11, or completing the same curriculum in a shorter amount of time. However, we cannot trace back which stressors might have ultimately fostered these results, as all of these potential causes are perfectly confounded with the reform (i.e., all changes happened simultaneously), we cannot disentangle their effects.

### Limitations

There are several limitations that are important to consider when interpreting the results of this study. These limitations include potential threats to internal and external validity. Regarding internal validity, it is important to consider that we used data from a cohort control design, whereby two representative cohorts of students from before the reform were compared with two representative cohorts of students after the reform. Although this cohort control design has been discussed as providing a good foundation for the investigation of intervention effects, as it resembles a natural experiment setting (Shadish et al., 2002), it might be possible that the cohorts already differed independent of the reform (e.g., due to historical events). In other words, we did not have a control group who did not receive the treatment at the same time that the students in the treatment group received the treatment (a difference in difference design; e.g., Cunningham, 2021). This of course provides a challenge for all research using reform data because reforms are typically implemented at the same time for all students in a specific state. Therefore, researchers are typically required to consider students from different states or cohorts within the same state (before the reform) as control groups, which in turn introduces different challenges and assumptions, particularly regarding their comparability. To address this potential limitation, we used survey weights to assure representativeness of the different cohorts. Notably, response rates on all assessments were 90% or larger at the student level (e.g., IEA, 2013). In addition, we inspected potential differences between the cohorts and specified adjusted models, in which we controlled for important (presumably relatively time-stable) covariates. All those checks suggested that if selection bias was present in our study, it should have been small at most (e.g., Hübner et al., 2017a).

Furthermore, it is important to underline that our findings are based on self-reports and that we did not have more objective markers to assess stress and health, for instance using data from health insurance agencies, medication records, or cortisol measures. Therefore, it cannot be ruled out that students, at least in part, also reported feeling more stressed because of ongoing discussions with their parents, friends from G9 cohorts, or the media. However, even if part of this effect could have been explained by these aspects, the remaining differences would have still remained of practical significance (e.g., Milde-Busch et al., 2010; Hübner et al., 2017a; Quis, 2018).

Regarding external validity, it is important to keep in mind that we considered representative data of one specific reform in one specific German state (Baden-Württemberg). Therefore, the findings should be generalized cautiously to discussions about effects of changes in instructional time. Most importantly, as shown in prior studies (Else-Quest et al., 2010; OECD, 2019), results on gender-related disparities are very heterogenous in STEM subjects across countries. The authors argue that one of the main drivers of gender differences are differential opportunity structures (e.g., equity in school enrollment). Based on this, it remains to be shown if our findings can be generalized to other countries where gender disparities are less or even more strongly pronounced, compared to Germany. However, doing this would require similar reforms to be implemented in other countries, which we are not aware of, even after consulting a large reform database (OECD, 2015). This also becomes evident when inspecting further related literature. Among others, findings on this topic are based on quite heterogeneous reforms (e.g., Allensworth et al., 2009; Domina et al., 2015; Huebener et al., 2017; Marcus et al., 2020), based on randomized controlled trials (e.g., Meyer and van Klaveren, 2013; Andersen et al., 2016) or cross-sectional secondary data analysis (e.g., Lavy, 2015). Before generalizing results from our study to the general debate about learning time or other environments (e.g., other states or reforms), researchers and practitioners should carefully consider potential similarities and differences.

Finally, the major change implemented by the G8-reform constitutes a school time compression, which was implemented by increasing average time per week spent in lower secondary school (Homuth, 2017). However, beyond these changes, other different elements changed simultaneously with the introduction of the G8-reform, for instance, educational standards were introduced and schools were required to develop a school-specific curriculum (Hübner et al., 2017a). Therefore, although the instructional time change is probably the most dominant feature of the reform, we cannot rule out that other changes might have affected our findings. Results of our study should therefore be interpreted cautiously as reform effects (e.g., a combination of different changes happening at the same time) rather than as pure effects of a change in instructional time.

## Conclusion

In this study, we investigated the gender-specific effects of an instructional school time reform on student achievement and motivation in STEM subjects, as well as on school-related stress and health. For most outcomes, we found substantial gender disparities favoring boys (e.g., in mathematics and physics), which did not intensify after the reform, but rather seemed to perpetuate. In contrast to subject-specific effects, significant gender × reform interaction effects were only evident on aspects of school-related stress and health, namely the Overload dimension of stress and the Overburdening and Achievement-related fear dimensions of health. From a more general standpoint our findings underscore the relevance of explicitly considering gender disparities when developing, implementing, and evaluating policy reforms.

## Data Availability Statement

Publicly available datasets were analyzed in this study. This data can be found here: this manuscript uses data from the National Educational Panel Study (NEPS): Additional Study Baden-Wuerttemberg, doi: 10.5157/NEPS:BW:3.2.0. From 2008 to 2013, NEPS data were collected as part of the Framework Program for the Promotion of Empirical Educational Research funded by the German Federal Ministry of Education and Research (BMBF). As of 2014, NEPS has been carried out by the Leibniz Institute for Educational Trajectories (LIfBi) at the University of Bamberg in cooperation with a nationwide network.

## Ethics Statement

The NEPS study is conducted under the supervision of the German Federal Commissioner for Data Protection and Freedom of Information (BfDI) and in coordination with the German Standing Conference of the Ministers of Education and Cultural Affairs (KMK) and – in the case of surveys at schools – the Educational Ministries of the respective Federal States. The studies involving human participants, including all data collection procedures, instruments, and documents, were reviewed and approved by the data protection unit of the Leibniz Institute for Educational Trajectories (LIfBi). Written informed consent to participate in this study was provided by the participants, if they were 18 years or older or the participants, and their legal guardian/next of kin, if they were below 18 years old (18 is the legal age of consent in Germany). The necessary steps are taken to protect participants’ confidentiality according to national and international regulations of data security. Participation in the NEPS study is voluntary and based on the informed consent of participants. This consent to participate in the NEPS study can be revoked at any time.

## Author Contributions

NH: conceptualization, formal analysis, writing—original draft, writing—review and editing, and project administration. WW, JM, and HW: conceptualization, writing—review and editing. All authors contributed to the article and approved the submitted version.

## Conflict of Interest

The authors declare that the research was conducted in the absence of any commercial or financial relationships that could be construed as a potential conflict of interest.

## Publisher’s Note

All claims expressed in this article are solely those of the authors and do not necessarily represent those of their affiliated organizations, or those of the publisher, the editors and the reviewers. Any product that may be evaluated in this article, or claim that may be made by its manufacturer, is not guaranteed or endorsed by the publisher.

## Footnotes

1
https://www.neps-data.de/Data-Center/Data-and-Documentation

## References

[B1] Adams R. J. (2005). Reliability as a measurement design effect. *Stud. Educ. Eval.* 31 162–172. 10.1016/j.stueduc.2005.05.008

[B2] Agnafors S. Barmark M. Sydsjö G. (2021). Mental health and academic performance: a study on selection and causation effects from childhood to early adulthood. *Soc. Psychiatry Psychiatr. Epidemiol.* 56 857–866. 10.1007/s00127-020-01934-5 32813024 PMC8068628

[B3] Allensworth E. Nomi T. Montgomery N. Lee V. E. (2009). College preparatory curriculum for all: academic consequences of requiring algebra and English I for ninth graders in Chicago. *Educ. Eval. Policy Anal.* 31 367–391. 10.3102/0162373709343471

[B4] Altuzarra A. Gálvez-Gálvez C. González-Flores A. (2021). Is gender inequality a barrier to economic growth:A panel data analysis of developing countries. *Sustainability* 13:367. 10.3390/su13010367

[B5] Andersen S. C. Humlum M. K. Nandrup A. B. (2016). Increasing instruction time in school does increase learning. *Proc. Natl. Acad. Sci. U.S.A.* 113 7481–7484. 10.1073/pnas.1516686113 27325778 PMC4941499

[B6] Arens A. K. Marsh H. W. Pekrun R. Lichtenfeld S. Murayama K. vom Hofe R. (2017). Math self-concept, grades, and achievement test scores: long-term reciprocal effects across five waves and three achievement tracks. *J. Educ. Psychol.* 109 621–634. 10.1037/edu0000163

[B7] Baumert J. Nagy G. Lehmann R. (2012). Cumulative advantages and the emergence of social and ethnic inequality: matthew effects in reading and mathematics development within elementary schools? *Child Dev.* 83 1347–1367. 10.1111/j.1467-8624.2012.01779.x 22616792

[B8] Bergmüller S. (2003). “Schulische Belastung und gesundheitliche Beschwerden [School-related demands and health problems],” in *PISA 2003*, eds Haider G. Reiter C. (Leykam: Internationaler Vergleich von Schülerleistungen).

[B9] Bergmüller S. (2007). *Schulstress Unter Jugendlichen: Entstehungsbedingungen, vermittelnde Prozesse und Folgen. Eine empirische Studie im Rahmen von PISA 2003 [School stress among adolescents: Originating conditions, mediating processes, and consequences. An empirical study in the context of PISA 2003].* Cham: Springer.

[B10] Berkowitz T. Schaeffer M. W. Maloney E. A. Peterson L. Gregor C. Levine S. C. (2015). Math at home adds up to achievement in school. *Science* 350 196–198. 10.1126/science.aac7427 26450209

[B11] Biewen M. Schwerter J. (2021). Does more maths and natural sciences in high school increase the share of female STEM workers? Evidence from a curriculum reform. *Appl. Econ..* 10.1080/00036846.2021.1983139 [Epub ahead of print]

[B12] Blossfeld H. P. Rossbach H. G. von Maurice J. (eds) (2011). *Education as a Lifelong Process - The German National Educational Panel Study (NEPS).* Wiesbaden: Springer VS.

[B13] Büttner B. Thomsen S. L. (2015). Are we spending too many years in school? Causal evidence of the impact of shortening secondary school duration. *German Econ. Rev.* 16 65–86. 10.1111/geer.12038

[B14] Cohen J. (1988). *Statistical Power Analysis for the Behavioral Sciences*, 2nd Edn. Mahwah, NJ: Lawrence Erlbaum Associates.

[B15] Cuban L. (2008). The perennial reform: fixing school time. *Phi Delta Kappan* 90 240–250. 10.1177/003172170809000404

[B16] Cunningham S. (2021). *Causal Inference: The Mixtape.* London: Yale University Press.

[B17] Denissen J. J. A. Zarrett N. R. Eccles J. S. (2007). I like to do it, I’m able, and I know I am: longitudinal couplings between domain-specific achievement, self-concept, and interest. *Child Dev.* 78 430–447. 10.1111/j.1467-8624.2007.01007.x 17381782

[B18] DiStefano C. Motl R. W. (2006). Further investigating method effects associated with negatively worded items on self-report surveys. *Struct. Equ. Model. Multidiscip. J.* 13 440–464. 10.1207/s15328007sem1303_6 26627889

[B19] Domina T. McEachin A. Penner A. Penner E. (2015). Aiming high and falling short. *Educ. Eval. Policy Anal.* 37 275–295. 10.3102/0162373714543685

[B20] Duchhardt C. (2015). *NEPS Technical Report for Mathematics: Scaling Results for the Additional Study Baden-Württemberg.* Bamberg: Leibniz-Institut für Bildungsverläufe e.V.

[B21] Eccles J. S. (1983). “Expectancies, values, and academic behaviors,” in *Achievement and Achievement Motivation*, ed. Spence J. (Dallas, TX: Freeman), 75–146.

[B22] Eccles J. S. Wigfield A. (2002). Motivational beliefs, values, and goals. *Annu. Rev. Psychol.* 53 109–132. 10.1146/annurev.psych.53.100901.135153 11752481

[B23] Eccles J. S. Wigfield A. (2020). From expectancy-value theory to situated expectancy-value theory: a developmental, social cognitive, and sociocultural perspective on motivation. *Contemp. Educ. Psychol.* 61:101859. 10.1016/j.cedpsych.2020.101859

[B24] Else-Quest N. M. Hyde J. S. Linn M. C. (2010). Cross-national patterns of gender differences in mathematics: a meta-analysis. *Psychol. Bull.* 136 103–127. 10.1037/a0018053 20063928

[B25] Enders C. K. (2010). *Applied Missing Data Analysis.* New York, NY: Guilford Press.

[B26] Fiorilli C. de Stasio S. Di Chiacchio C. Pepe A. Salmela-Aro K. (2017). School burnout, depressive symptoms and engagement: their combined effect on student achievement. *Int. J. Educ. Res.* 84 1–12. 10.1016/j.ijer.2017.04.001

[B27] Frenzel A. C. Goetz T. Pekrun R. Watt H. M. G. (2010). Development of mathematics interest in adolescence: influences of gender, family, and school context. *J. Res. Adolesc.* 20 507–537. 10.1111/j.1532-7795.2010.00645.x

[B28] Haberkorn K. Pohl S. (2013). *Cognitive basic skills – Data in the Scientific Use File.* Bamberg: University of Bamberg.

[B29] Hammond A. Matulevich E. R. Beegle K. Kumaraswamy S. K. (2020). *The Equality Equation: Advancing the Participation of Women and Girls in STEM.* Washington, DC: The World Bank.

[B30] Hampel P. Petermann F. (2006). Perceived stress, coping, and adjustment in adolescents. *J. Adolesc. Health* 38 409–415. 10.1016/j.jadohealth.2005.02.014 16549302

[B31] Hjern A. Alfven G. Östberg V. (2008). School stressors, psychological complaints and psychosomatic pain. *Acta Paediatr.* 97 112–117. 10.1111/j.1651-2227.2007.00585.x 18076714

[B32] Homuth C. (2017). *Die G8-Reform in Deutschland [The G8-reform in Germany].* Berlin: Springer.

[B33] Hu L. Bentler P. M. (1999). Cutoff criteria for fit indexes in covariance structure analysis: conventional criteria versus new alternatives. *Struct. Equ. Model. Multidiscip. J.* 6 1–55. 10.1080/10705519909540118

[B34] Hübner N. Rieger S. Wagner W. (2016a). *NEPS Technical Report for Biological Competence: Scaling Results for the Additional Study Baden-Wuerttemberg (NEPS Survey Paper No. 9).* Bamberg: Leibniz Institute for Educational Trajectories.

[B35] Hübner N. Rieger S. Wagner W. (2016b). *NEPS Technical Report for Physics Competence: Scaling Results for the Additional Study Baden-Wuerttemberg (NEPS Survey Paper No. 11).* Bamberg: Leibniz Institute for Educational Trajectories.

[B36] Hübner N. Wagner W. Hochweber J. Neumann M. Nagengast B. (2020). Comparing apples and oranges: curricular intensification reforms can change the meaning of students’ grades! Curricular intensification reforms can change the meaning of students’ grades! *J. Educ. Psychol.* 112 204–220. 10.1037/edu0000351

[B37] Hübner N. Wagner W. Kramer J. Nagengast B. Trautwein U. (2017a). Die G8-Reform in Baden-Württemberg: kompetenzen, Wohlbefinden und Freizeitverhalten vor und nach der Reform: kompetenzen, Wohlbefinden und Freizeitverhalten vor und nach der Reform [The G8 reform in Baden-Württemberg: competencies, wellbeing and leisure time before and after the reform]. *Z. Erziehungswissenschaft* 20 748–771. 10.1007/s11618-017-0737-3

[B38] Hübner N. Wille E. Cambria J. Oschatz K. Nagengast B. Trautwein U. (2017b). Maximizing gender equality by minimizing course choice options? Effects of obligatory coursework in math on gender differences in STEM. *J. Educ. Psychol.* 109 993–1009. 10.1037/edu0000183

[B39] Hübner N. Wagner W. Nagengast B. Trautwein U. (2019). Putting all students in one basket does not produce equality: gender-specific effects of curricular intensification in upper secondary school. *Sch. Effect. Sch. Improv.* 30 261–285. 10.1080/09243453.2018.1504801

[B40] Huebener M. Kuger S. Marcus J. (2017). Increased instruction hours and the widening gap in student performance. *Labour Econ.* 47 15–34. 10.1016/j.labeco.2017.04.007

[B41] IEA (2013). *Methodenbericht: NEPS Zusatzstudie zur G8-Reform in Baden-Württemberg: Haupterhebung - Frühjahr 2011 (A72) [Technical report: NEPS additional study on the G8 reform in Baden-Württemberg.* Cham: Spring.

[B42] Jacobs J. E. Simpkins S. D. (2005). Mapping leaks in the math, science, and technology pipeline. *New Dir. Child Adolesc. Dev.* 110 3–6. 10.1002/cd.145 16509535

[B43] Jöreskog K. G. Goldberger A. S. (1975). Estimation of a model with multiple indicators and multiple causes of a single latent variable. *J. Am. Stat. Assoc.* 70 631–639. 10.1080/01621459.1975.10482485

[B44] Kühn S. M. van Ackeren I. Bellenberg G. Reintjes C. Im Brahm G. (2013). Wie viele Schuljahre bis zum Abitur? Eine multiperspektivische Standortbestimmung im Kontext der aktuellen Schulzeitdebatte [How many years until abitur in German upper secondary schooling? – Taking stock in the context of current school duration debates]. *Z. Erziehungswissenschaft* 16 115–136. 10.1007/s11618-013-0339-7

[B45] Lavy V. (2015). Do differences in schools’ instruction time explain international achievement gaps? Evidence from developed and developing countries. *Econ. J.* 125 F397–F424. 10.1111/ecoj.12233

[B46] Lazarides R. Dicke A. L. Rubach C. Eccles J. S. (2020). Profiles of motivational beliefs in math: exploring their development, relations to student-perceived classroom characteristics, and impact on future career aspirations and choices. *J. Educ. Psychol.* 112 70–92. 10.1037/edu0000368

[B47] Lazarides R. Lauermann F. (2019). Gendered paths into STEM-related and language-related careers: girls’ and boys’ motivational beliefs and career plans in math and language arts. *Front. Psychol.* 10:1243. 10.3389/fpsyg.2019.01243 31244713 PMC6563766

[B48] Ma X. Johnson W. (2008). “Mathematics as the critical filter: curricular effects on gendered career choices,” in *Gender and Occupational Outcomes: Longitudinal Assessments of Individual, Social, and Cultural Influences*, eds Watt H. M. G. Eccles J. S. (Washington, DC: American Psychological Association), 55–83.

[B49] MacCallum R. C. Browne M. W. Sugawara H. M. (1996). Power analysis and determination of sample size for covariance structure modeling. *Psychol. Methods* 1 130–149. 10.1037/1082-989X.1.2.130

[B50] Maceira H. (2017). Economic benefits of gender equality in the EU. *Intereconomics* 52 178–183. 10.1007/s10272-017-0669-4

[B51] Makarova E. Aeschlimann B. Herzog W. (2019). The gender gap in STEM fields: the impact of the gender stereotype of math and science on secondary students’ career aspirations. *Front. Educ.* 4:60. 10.3389/feduc.2019.00060

[B52] Marcus J. Reif S. Wuppermann A. Rouche A. (2020). Increased instruction time and stress-related health problems among school children. *J. Health Econ.* 70:102256. 10.1016/j.jhealeco.2019.102256 32028089

[B53] Marsh H. W. (1990). The structure of academic self-concept: the Marsh/Shavelson model. *J. Educ. Psychol.* 82 623–636. 10.1037/0022-0663.82.4.623

[B54] Marsh H. W. (1992). *Self Description Questionnaire (SDQ) III: A Theoretical and Empirical Basis for the Measurement of Multiple Dimensions of Late Adolescent Self-Concept: A Test Manual and a Research Monograph.* San Antonio, TX: The Psychological Corporation.

[B55] Marsh H. W. Craven R. G. (2006). Reciprocal effects of self-concept and performance from a multidimensional perspective: beyond seductive pleasure and unidimensional perspectives. *Perspect. Psychol. Sci.* 1 133–163. 10.1111/j.1745-6916.2006.00010.x 26151468

[B56] Marsh H. W. Martin A. J. Yeung A. S. Craven R. (2016). “Competence self-perceptions,” in *Handbook of Competence and Motivation*, eds Elliot A. J. Dweck C. S. Yeager D. (New York, NY: Guilford Press), 85–115.

[B57] Marsh H. W. Morin A. J. S. Parker P. D. Kaur G. (2014). Exploratory structural equation modeling: an integration of the best features of exploratory and confirmatory factor analysis. *Annu. Rev. Clin. Psychol.* 10 85–110. 10.1146/annurev-clinpsy-032813-153700 24313568

[B58] Marsh H. W. O’Neill R. (1984). Self description questionnaire III: the construct validity of multidimensional self-concept ratings by late adolescents. *J. Educ. Meas.* 21 153–174. 10.1111/j.1745-3984.1984.tb00227.x

[B59] McNeish D. Stapleton L. M. Silverman R. D. (2017). On the unnecessary ubiquity of hierarchical linear modeling. *Psychol. Methods* 22 114–140. 10.1037/met0000078 27149401

[B60] Meinck S. Brese F. (2020). *Gender Gaps in Science are Not a Given: Evidence on International Trends in Gender Gaps in Science Over 20 Years: Compass: Briefs in Education No. 11.* Paris: IEA.

[B61] Mejía-Rodríguez A. M. Luyten H. Meelissen M. R. M. (2021). Gender differences in mathematics self-concept across the world: an exploration of student and parent data of TIMSS 2015. *Int. J. Sci. Math. Educ.* 19 1229–1250. 10.1007/s10763-020-10100-x

[B62] Merton R. K. (1968). The Matthew effect in science. *Science* 159 56–63. 10.1126/science.159.3810.56 5634379

[B63] Meyer E. van Klaveren C. (2013). The effectiveness of extended day programs: evidence from a randomized field experiment in the Netherlands. *Econ. Educ. Rev.* 36 1–11. 10.1016/j.econedurev.2013.04.002

[B64] Meyer T. Schneider H. Thomsen S. L. (2019). New evidence on the effects of the shortened school duration in the German states: an evaluation of post-secondary education decisions. *German Econ. Rev.* 20 e201–e253. 10.1111/geer.12162

[B65] Milde-Busch A. Blaschek A. Borggräfe I. von Kries R. Straube A. Heinen F. (2010). Besteht ein Zusammenhang zwischen der verkürzten Gymnasialzeit und Kopfschmerzen und gesundheitlichen Belastungen bei Schülern im Jugendalter? [Is there an association between the reduced school years in grammar schools and headache and other health complaints in adolescent students?]. *Klinische Pädiatrie* 222 255–260. 10.1055/s-0030-1252012 20455196

[B66] Moksnes U. K. Moljord I. E. Espnes G. A. Byrne D. G. (2010). The association between stress and emotional states in adolescents: the role of gender and self-esteem. *Pers. Individ. Diff.* 49 430–435. 10.1016/j.paid.2010.04.012

[B67] Muth L. K. Muth B. O. (1998-2017). *Mplus User’s Guide*, Eighth Edn. Los Angeles, CA: Muthén & Muthén.

[B68] Nagy G. Watt H. M. G. Eccles J. S. Trautwein U. Lüdtke O. Baumert J. (2010). The development of students’ mathematics self-concept in relation to gender: different countries, different trajectories? *J. Res. Adolesc.* 20 482–506. 10.1111/j.1532-7795.2010.00644.x

[B69] Nomi T. Allensworth E. (2009). “Double-Dose” algebra as an alternative strategy to remediation: effects on students’ academic outcomes. *J. Res. Educ. Effect.* 2 111–148. 10.1080/19345740802676739

[B70] OECD (2015). *Education Policy Outlook 2015.* Paris: OECD.

[B71] OECD (2019). *PISA 2018 Results (Volume II): Where All Students Can Succeed.* Paris: OECD Publishing.

[B72] Oehlert G. W. (1992). A note on the delta method. *Am. Stat.* 46 27–29. 10.1080/00031305.1992.10475842

[B73] Parker P. D. Schoon I. Tsai Y. M. Nagy G. Trautwein U. Eccles J. S. (2012). Achievement, agency, gender, and socioeconomic background as predictors of postschool choices: a multicontext study. *Dev. Psychol.* 48 1629–1642. 10.1037/a0029167 22799584

[B74] Parker P. D. van Zanden B. Marsh H. W. Owen K. Duineveld J. J. Noetel M. (2020). The intersection of gender, social class, and cultural context: a meta-analysis. *Educ. Psychol. Rev.* 32 197–228. 10.1007/s10648-019-09493-1

[B75] Patall E. A. Cooper H. Allen A. B. (2010). Extending the school day or school year. *Rev. Educ. Res.* 80 401–436. 10.3102/0034654310377086

[B76] Pischke J. S. (2007). The impact of length of the school year on student performance and earnings: evidence from the German short school years. *Econ. J.* 117 1216–1242. 10.1111/j.1468-0297.2007.02080.x

[B77] Quis J. S. (2018). Does compressing high school duration affect students’ stress and mental health? Evidence from the national educational panel study. *J. Econ. Stat.* 238 441–476. 10.1515/jbnst-2018-0004

[B78] Rawson K. Stahovich T. F. Mayer R. E. (2017). Homework and achievement: using smartpen technology to find the connection. *J. Educ. Psychol.* 109 208–219. 10.1037/edu0000130

[B79] Salmela-Aro K. Tynkkynen L. (2012). Gendered pathways in school burnout among adolescents. *J. Adolesc.* 35 929–939. 10.1016/j.adolescence.2012.01.001 22300678

[B80] Santos Silva M. Klasen S. (2021). Gender inequality as a barrier to economic growth: a review of the theoretical literature. *Rev. Econ. Household* 19 581–614. 10.1007/s11150-020-09535-6

[B81] Scheerens J. (ed.) (2014). *Effectiveness of Time Investments in Education.* Berlin: Springer International Publishing.

[B82] Schönberger B. Aßmann C. (2014). *Weighting the Additional Study in Baden-Wuerttemberg of the National Educational Panel Study.* Bamberg: University of Bamberg.

[B83] Schoon I. Eccles J. S. (eds) (2014). *Gender Differences in Aspirations and Attainment: A Life Course Perspective.* Cambridge, MA: Cambridge University Press.

[B84] Seaton M. Marsh H. W. Parker P. D. Craven R. G. Yeung A. S. (2015). The reciprocal effects model revisited. *Gift. Child Q.* 59 143–156. 10.1177/0016986215583870

[B85] Shadish W. R. Cook T. D. Campbell D. T. (2002). *Experimental and Quasi-Experimental Designs for Generalized Causal Inference.* Boston, MA: Houghton Mifflin.

[B86] Shavelson R. J. Hubner J. J. Stanton G. C. (1976). Self-concept: validation of construct interpretations. *Rev. Educ. Res.* 46 407–441. 10.3102/00346543046003407

[B87] Stanat P. Schipolowski S. Rjosk C. Weirich S. Haag N. (2017). *IQB Trends in Student Achievement 2016: The Second National Assessment of German and Mathematics Proficiencies at the End of Fourth Grade.* Münster: Waxmann.

[B88] Trautwein U. Lüdtke O. Marsh H. W. Köller O. Baumert J. (2006). Tracking, grading, and student motivation: using group composition and status to predict self-concept and interest in ninth-grade mathematics. *J. Educ. Psychol.* 98 788–806. 10.1037/0022-0663.98.4.788

[B89] Trautwein U. Neumann M. Nagy G. Lüdtke O. Maaz K. (2010). “Institutionelle Reformen und individuelle Entwicklung: hintergrund und Fragestellung der Studie TOSCA-Repeat [Institutional reforms and individual development: background and research question of the TOSCA-Repeat study],” in *Schulleistungen von Abiturienten (1st ed.)*, eds Trautwein U. Neumann M. Nagy G. Lüdtke O. Maaz K. (Berlin: Springer).

[B90] Tuominen-Soini H. Salmela-Aro K. (2014). Schoolwork engagement and burnout among Finnish high school students and young adults: profiles, progressions, and educational outcomes. *Dev. Psychol.* 50 649–662. 10.1037/a0033898 23895174

[B91] United Nations General Assembly (1948). *Universal Declaration of Human Rights.* New York, NY: United Nations General Assembly.

[B92] Updegraff K. A. Eccles J. S. Barber B. L. O’brien K. M. (1996). Course enrollment as self-regulatory behavior: who takes optional high school math courses? *Learn. Individ. Diff.* 8 239–259. 10.1016/S1041-6080(96)90016-3

[B93] van Sonderen E. Sanderman R. Coyne J. C. (2013). Ineffectiveness of reverse wording of questionnaire items: let’s learn from cows in the rain. *PLoS One* 8:e68967. 10.1371/journal.pone.0068967 23935915 PMC3729568

[B94] Wagner P. Schober B. Spiel C. (2008). Time students spend working at home for school. *Learn. Instr.* 18 309–320. 10.1016/j.learninstruc.2007.03.002

[B95] Watt H. M. G. (2004). Development of adolescents’ self-perceptions, values, and task perceptions according to gender and domain in 7th- through 11th-grade Australian students. *Child Dev.* 75 1556–1574. 10.1111/j.1467-8624.2004.00757.x 15369531

[B96] Watt H. M. G. Bucich M. Dacosta L. (2019). Adolescents’ motivational profiles in mathematics and science: associations with achievement striving, career aspirations and psychological wellbeing. *Front. Psychol.* 10:990. 10.3389/fpsyg.2019.00990 31316409 PMC6610331

[B97] Watt H. M. G. Hyde J. S. Petersen J. Morris Z. A. Rozek C. S. Harackiewicz J. M. (2017). Mathematics—a critical filter for STEM-related career choices? A longitudinal examination among Australian and U.S. adolescents. *Sex Roles* 77 254–271. 10.1007/s11199-016-0711-1

[B98] Watt H. M. G. Shapka J. D. Morris Z. A. Durik A. M. Keating D. P. Eccles J. S. (2012). Gendered motivational processes affecting high school mathematics participation, educational aspirations, and career plans: a comparison of samples from Australia, Canada, and the United States. *Dev. Psychol.* 48 1594–1611. 10.1037/a0027838 22468566

[B99] Widlund A. Tuominen H. Korhonen J. (2018). Academic well-being, mathematics performance, and educational aspirations in lower secondary education: changes within a school year. *Front. Psychol.* 9:297. 10.3389/fpsyg.2018.00297 29593603 PMC5859340

[B100] Wu H. Guo Y. Yang Y. Le Z. Guo C. (2021). A meta-analysis of the longitudinal relationship between academic self-concept and academic achievement. *Educ. Psychol. Rev..* 10.1007/s10648-021-09600-1 [Epub ahead of print]

[B101] Yu C. Y. (2002). *Evaluating Cutoff Criteria of Model Fit Indices for Latent Variable Models with Binary and Continuous Outcomes: A Dissertation Submitted in Partial Satisfaction of the Requirements for the Degree. Doctor of Philosophy in Education.* Los Angeles, CA: University of California.

[B102] Zhang X. Noor R. Savalei V. (2016). Examining the effect of reverse worded items on the factor structure of the need for cognition scale. *PLoS One* 11:e0157795. 10.1371/journal.pone.0157795 27305001 PMC4909292

