# Supplementary material for: To Those Who Have, More Will Be Given? Effects of an Instructional Time Reform on Gender Disparities in STEM Subjects, Stress, and Health
Source: Front Psychol. 2022 Feb 21;13:816358. doi: 10.3389/fpsyg.2022.816358 (PMC8899205; doi:10.3389/fpsyg.2022.816358)
Supplement: Supplementary file 1 [file Data_Sheet_1.docx]

# Supplemental Material

# Table 1

Stress Items

| Construct: Stress | |
| --- | --- |
| Statement: How strongly do the following statements apply to you? | |
| Item | Wording |
| t5a | I am tense when I get home from school. |
| t5b | Sometimes I have trouble falling asleep because problems from school are on my mind. |
| t5c | It happens that I react irritably when others start talking to me about school. |
| t5d | I feel that school is overwhelming me. |
| t5e | Even during my free time I think about troubles at school. |
| t5f | I consider the requirements at school in general as stressful. |
| t5g | After school I am often exhausted. |
| t5h | Thinking of school makes me feel uncomfortable. |
| t5i | Pressure at school is too high. |
| t5j | School is eating me up. |
| t5k | It is hard for me to conciliate school with other obligations. |
| t5l | School often makes me feel tired andexhausted. |
| t5m | It is easy for me to recover from school during my free time. |
| t5n | I can relax well during my free time. |
| t5o | Apart from school, I do not have time for anything else. |

*Note.*Items t5m and t5n were dropped from the analysis because they showed low item-total correlation. Items were answered on a scale from 1 (*completely disagree*) to 4 (*completely agree*). The full questionnaire can be downloaded here: https://www.neps-data.de/Portals/0/NEPS/Datenzentrum/Forschungsdaten/BW/2-0-0/BW_2-0-0_Q_en.pdf

# Table 2

Health Items

| Construct: Health | |
| --- | --- |
| Statement: How often have you had the following problems in the last 6 weeks? | |
| Item | Wording |
| t54a | Nervousness, inner anxiety |
| t54b | Headaches |
| t54c | Strong heart palpitations |
| t54d | Fear that it’s all getting too much |
| t54e | Difficulty concentrating |
| t54f | Sleep disturbances |
| t54g | Bad dreams |
| t54h | Excessive sweating |
| t54i | Vomiting |
| t54j | Easily irritable |
| t54k | Feelings of dizziness |
| t54l | Tiredness, fatigue |
| t54m | Incapable of relaxing |
| t54n | Severe forgetfulness, distraction |
| t54o | Angry at everything |
| t54p | Feeling of being worthless |
| t54q | Fear of going to school |
| t54r | Shakiness, weakness |
| t54s | Nausea |
| t54t | Loss of appetite |
| t54u | Backache |
| t54v | Sadness |
| t54w | Feeling that excessive demands are being made of me |
| t54x | Eating binges |
| t54y | Feeling of inner emptiness |
| t54z | Stomach ache |

*Note.*Items were answered on a scale from 1 (*never*) to 4 (*more than 6 times*). The full questionnaire can be downloaded here: https://www.neps-data.de/Portals/0/NEPS/Datenzentrum/Forschungsdaten/BW/2-0-0/BW_2-0-0_Q_en.pdf

# Table 3

Factor Loadings in the Multiple Group Exploratory SEM for Stress

| Item/Dimension | Difficulties to relax | Exhaustion | Overload | Malaise | Alignment issues |
| --- | --- | --- | --- | --- | --- |
| t5a | **0.29** | **0.29** | **-0.04** | 0.01 | **0.04** |
| t5b | **0.55** | 0.04 | 0.03 | 0.01 | 0.00 |
| t5c | **0.32** | 0.01 | 0.00 | **0.36** | -0.01 |
| t5d | **0.14** | **-0.04** | **0.38** | **0.08** | -0.01 |
| t5e | **0.38** | 0.00 | **0.19** | -0.02 | **0.04** |
| t5f | 0.02 | **0.16** | **0.50** | 0.02 | -0.02 |
| t5g | 0.01 | **0.80** | **0.06** | **-0.03** | **-0.03** |
| t5h | -0.01 | 0.03 | 0.11 | **0.58** | 0.01 |
| t5i | **-0.03** | **0.05** | **0.56** | 0.01 | **0.05** |
| t5j | **0.04** | -0.02 | **0.24** | **0.17** | **0.13** |
| t5k | -0.01 | **0.11** | -0.01 | **0.09** | **0.43** |
| t5l | 0.01 | **0.67** | 0.00 | **0.14** | **0.06** |
| t5o | **0.06** | -0.02 | 0.06 | **-0.09** | **0.40** |

*Note*. Model fit for the unadjusted model amounted to χ2(520) = 833.378, *p*< .001, CFI = .99, TLI = .98, RMSEA = .03, SRMR = .03. Items t5m and t5n were dropped from the analysis because they showed low item-total correlation. *p* values <.05 are printed in bold. The three items with the highest loading per factor areshaded in grey.

# Table 4

Factor Loadings in the Multiple Group Exploratory SEM for Health

| Item/  Dimension | Overburdening | Achievement-related fear | Diverse symptoms | Uneasiness | Depressive symptoms | Gastrointestinal issues |
| --- | --- | --- | --- | --- | --- | --- |
| t54a | **0.19** | **0.27** | **0.05** | **0.14** | 0.01 | -0.01 |
| t54b | **0.16** | 0.02 | **0.27** | 0.00 | -0.01 | **0.14** |
| t54c | 0.06 | **0.13** | 0.00 | **0.30** | **-0.05** | 0.03 |
| t54d | -0.02 | **0.63** | **0.04** | 0.01 | 0.01 | -0.02 |
| t54e | **0.47** | **0.11** | -0.01 | **0.10** | -0.01 | 0.00 |
| t54f | **0.14** | 0.01 | 0.19 | **0.26** | 0.07 | -0.01 |
| t54g | 0.00 | 0.02 | **0.23** | **0.22** | **0.10** | -0.03 |
| t54h | 0.06 | 0.00 | -0.03 | **0.26** | -0.01 | 0.04 |
| t54i | -0.01 | 0.00 | **-0.06** | 0.01 | 0.00 | **0.19** |
| t54j | **0.47** | 0.01 | **0.10** | **-0.07** | **0.16** | 0.00 |
| t54k | 0.01 | -0.01 | **0.16** | **0.16** | 0.02 | **0.20** |
| t54l | **0.52** | **0.05** | 0.05 | -0.03 | -0.02 | 0.03 |
| t54m | **0.18** | **0.22** | 0.08 | **0.17** | 0.04 | -0.03 |
| t54n | **0.43** | -0.01 | **-0.07** | **0.16** | **0.06** | 0.01 |
| t54o | **0.27** | **0.09** | **-0.04** | 0.01 | **0.29** | 0.02 |
| t54p | **-0.06** | 0.04 | -0.01 | 0.01 | **0.51** | **0.03** |
| t54q | -0.03 | **0.32** | **-0.09** | **0.11** | 0.02 | **0.05** |
| t54r | **-0.07** | 0.05 | 0.05 | **0.24** | 0.03 | **0.11** |
| t54s | 0.02 | 0.00 | -0.02 | -0.03 | 0.01 | **0.57** |
| t54t | 0.01 | 0.01 | 0.03 | **0.07** | **0.13** | **0.19** |
| t54u | **0.23** | -0.01 | **0.18** | 0.06 | -0.04 | **0.10** |
| t54v | **0.05** | **0.02** | **0.07** | -0.04 | **0.55** | **0.03** |
| t54w | **0.07** | **0.61** | -0.03 | **-0.04** | 0.02 | 0.02 |
| t54x | **0.24** | -0.02 | 0.05 | 0.06 | **0.12** | 0.00 |
| t54y | 0.04 | -0.02 | -0.02 | **0.07** | **0.57** | **-0.03** |
| t54z | 0.01 | 0.02 | **0.21** | 0.01 | 0.01 | **0.28** |

*Note*. Model fit for the unadjusted model amounted to χ^2^(2452) = 4041.463, *p*< .001, CFI = .95, TLI = .95, RMSEA = .03, SRMR = .04. *p* values <.05 are printed in bold. The three items with the highest loading per factor areshaded in grey.

# Table 5

Course-Level Enrollment by Gender in Percentages

|  | Male | Female |
| --- | --- | --- |
| Mathematics |  |  |
| Advanced course level | 100% | 100% |
| English |  |  |
| Advanced course level | 84.3% | 83.9% |
| Basic course level | 15.7% | 16.1% |
| Physics |  |  |
| Advanced course level | 29.9% | 8.7% |
| Basic course level | 27.7% | 31% |
| De-selection | 42.4% | 60.3% |
| Biology |  |  |
| Advanced course level | 34.4% | 41% |
| Basic course level | 31.2% | 39.4% |
| De-selection | 34.4% | 19.6% |

*Note.* All students were required to enroll in advanced math courses (e.g., Hübner et al., 2020; Hübner, Wille, et al., 2017). De-selection means that students did not enroll in this course on basic or advanced course level during upper secondary school.
